# Break-before-Make CMOS Inverter for Power-Efficient Delay Implementation

**DOI:** 10.1155/2014/349131

**Published:** 2014-11-26

**Authors:** Janez Puhan, Dušan Raič, Tadej Tuma, Árpád Bűrmen

**Affiliations:** Faculty of Electrical Engineering, University of Ljubljana, Tržaška 25, 1000 Ljubljana, Slovenia

## Abstract

A modified static CMOS inverter with two inputs and two outputs is proposed to reduce short-circuit current in order to increment delay and reduce power overhead where slow operation is required. The circuit is based on bidirectional delay element connected in series with the PMOS and NMOS switching transistors. It provides differences in the dynamic response so that the direct-path current in the next stage is reduced. The switching transistors are never ON at the same time. Characteristics of various delay element implementations are presented and verified by circuit simulations. Global optimization procedure is used to obtain the most power-efficient transistor sizing. The performance of the modified CMOS inverter chain is compared to standard implementation for various delays. The energy (charge) per delay is reduced up to 40%. The use of the proposed delay element is demonstrated by implementing a low-power delay line and a leading-edge detector cell.

## 1. Introduction

Serial connection of inverters is often used for implementing low-precision delay in digital systems. However, the delay of cascaded inverters is power efficient only for small delays, which leads to an excessive power loss when longer delays are required. Each inverter in the chain drains additional parasitic energy that is approximately equal to the dynamic energy required for changing its input. Often the number of delay stages in a chain is reduced at the expense of increased node capacitances as long as capacitive loads do not introduce excessive direct-path energy.

Direct-path current is a well-known source of internal dynamic power consumption in CMOS logic. In well-designed circuits, it is estimated to be less than 20% of the dynamic dissipation [1] but may prohibitively increase in circuits with significant capacitive loads. The problem is efficiently solved if NMOS and PMOS gates of the CMOS inverter are driven by separate, time-skewed signals. This solution has been applied for large capacitive loads in [2] and later in [3–5]. All of these circuits have additional driving stages inserted in front of the split inverter inputs. The overhead of the additional components in terms of area and power consumption is justified only if it is outweighed by the savings obtained in the driving stages of large capacitive loads. On the gate-level logic, the overhead is hardly justified since the loads are small. Other gate-level techniques have also been proposed with the aim of reducing internal static power due to leakage currents in nanometer technologies [6, 7]. These techniques come with an area overhead and do not improve internal dynamic power.

**Figure 1:**
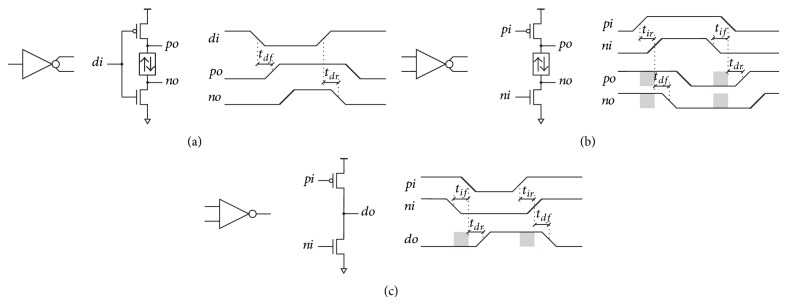
BBM inverter structures with time domain responses: (a) CMOS to BBM converter, (b) BBM inverter, and (c) BBM to CMOS converter.

The solution proposed in Figure 1 addresses the internal dynamic power consumption problem by inserting a bidirectional delay element at the inverter output to provide time-skewed signals for the next split-input inverter stage. The proposed structure provides break-before-make (BBM) switching with very low component overhead. No additional stages are needed. The overhead is low enough for the circuit to be used with small loads that are common in gate-level circuit design.

## 2. Circuit Operation

Input signal is transformed into two time-skewed signals by CMOS to BBM converter in Figure 1(a). At high to low transition of the input signal* di* both transistors switch, the PMOS opens and the NMOS goes into high-impedance state. The PMOS pulls the output node *po* to logical high. The delay *t*_*df*_ is defined by the PMOS transistor. The output node *no* follows with delay defined by the bidirectional delay element. Similar process is repeated in the opposite direction at low to high transition of *di*. Output signal pair *po*, *no* of the CMOS to BBM converter is used as input time-skewed signals *pi*, *ni* for the proposed inverter in Figure 1(b).

The proposed inverter is composed of a serially connected PMOS transistor, a bidirectional delay element, and a NMOS transistor. Circuit operation is best explained by an ideal transport delay timing diagram (Figure 1(b) right). The inverter input and output signals are applied as signal pairs *pi*, *ni* and *po*, *no*, respectively. From the point of view of static signals, the two signals in a signal pair represent the same logical level. Because of the built-in delay, they never change simultaneously. The first transition also referred to as* isolation* (nonbold slope in timing diagram in Figure 1(b)) is followed by the second transition also referred to as* information* (bold slope in timing diagram in Figure 1(b)). The isolation slope always precedes the information slope in any logical transition. Isolation time *t*_*i*_ is the time interval between the isolation and the information. During the isolation time, the inverter output is in a high-impedance state (indicated by the grayed output signal areas in timing diagram in Figure 1(b)), preserving the old logical state on capacitive load and preventing the direct-path current from flowing between the power supply and the ground.

Let us first assume that the input nodes *pi* and *ni* change from logical low to logical high, as presented in timing diagram in Figure 1(b). The voltage on node *pi* rises first, representing the isolation slope. The information slope at node *ni* follows after the rising isolation time *t*_*ir*_. The isolation slope switches the PMOS transistor into a high-impedance state. The old logical state is preserved on a capacitive load until the information slope opens the NMOS transistor and pulls the output node *no* to logical low. Because of the bidirectional delay element, the output node *po* transits to logical low later than node *no* and the state switching transient is complete. A similar process takes place when *pi* and *ni* transits from logical high to logical low.

The input isolation times are reproduced at the output thus allowing cascaded operation. Time skewing between input and output nodes of serially connected inverters is therefore guaranteed throughout the whole cascade.

Now, let us assume that one or both isolation times *t*_*ir*_, *t*_*if*_ are increased. This increases the time during which both transistors are in a high-impedance state but do not affect the skew between the output signals. The later depends only on the bidirectional delay element. Isolation times of serially connected inverters therefore do not accumulate from stage to stage. If the isolation time is reduced, the circuit functionality is maintained until *t*_*ir*_ or *t*_*if*_ becomes zero, and the circuit operation becomes identical to that of a standard CMOS inverter. Generally, the isolation slopes are defined by the inverter transistors, while the information slopes are defined by the delay element.

The role of input signals differs depending on the transition. Isolation slope is the falling edge at the NMOS gate or the rising edge at the PMOS gate. Similarly, the rising edge at the NMOS gate and the falling edge at the PMOS gate represent information slope. Because of dual input and output signals, the standard timing parameters must be redefined. Delay times *t*_*dr*_ and *t*_*df*_ are defined between input information slope and output isolation slope as presented in timing diagram in Figure 1(b). Standard rise and fall time definitions (10% to 90% and vice versa) apply to each signal separately. Isolation times *t*_*ir*_ and *t*_*if*_ are defined as the delay between the isolation slope and the corresponding information slope.

Time-skewed input signals are merged back into a single output by BBM to CMOS converter in Figure 1(c). An isolation falling slope on *ni* is followed by the information falling slope on *pi*. The first switches the NMOS transistor into a high-impedance state. Low logical state on output *do* is preserved on capacitive load until the information slope opens the PMOS transistor. During the isolation time *t*_*if*_, the converter output is in a high-impedance state. The PMOS pulls the output node *do* to logical high with delay *t*_*df*_. Similar process is going on at rising isolation and information slopes.

The propagation delay *T*_*p*_ is defined as the average of the low-to-high (*t*_*dLH*⁡_) and the high-to-low (*t*_*d*HL_) transition. For BBM inverter (Figure 1(b)), *t*_*dLH*⁡_ = *t*_*ir*_ + *t*_*df*_ and *t*_*d*HL_ = *t*_*if*_ + *t*_*dr*_. For a symmetric circuit with *t*_*dr*_ = *t*_*df*_ = *t*_*d*_ and *t*_*ir*_ = *t*_*if*_ = *t*_*i*_, the propagation delay is given by

(1) $$\begin{array}{c} T_{p}=\frac{t_{ir}+t_{df}+t_{if}+t_{dr}}{2}=t_{d}+t_{i}. \end{array}$$

Assuming that *t*_*d*_ is proportional to the equally sized standard CMOS inverter propagation delay (*T*_*p*,CMOS_), the following linear relation can be obtained:

(2) $$\begin{array}{c} T_{p}=k_{1}T_{p,\text{CMOS}}+t_{i}. \end{array}$$

By definition, *T*_*p*,CMOS_ is the average of the rise and the fall delay (*t*_*dLH*⁡,CMOS_ and *t*_*d*HL,CMOS_) of a CMOS inverter. Eliminated direct path in the proposed inverter provides more switching current for charging and discharging the capacitive load represented by the next stage. Therefore, *t*_*d*_ < *t*_*d*,CMOS_ and *k*_1_ < 1.

## 3. Charge Delay Analysis

Circuit power efficiency is measured by the power-delay product (PDP) and energy-delay product (EDP = PDP*T*_*p*_) [8, 9]. PDP corresponds to the energy required for one gate switch. EDP, on the other hand, represents a trade-off between energy and performance. Usually, PDP and EDP should be as low as possible thus resulting in minimum delay at minimal possible energy consumption.

When designing a low-precision delay, the situation is turned upside down. The goal is to implement the required delay *T*_*p*_ at lowest possible energy consumption. EDP can be reduced by reducing the supply voltage or the charge. Because the supply voltage cannot be changed, this means that we are looking for minimal charge *Q* required for implementing delay *T*_*p*_.

**Figure 2:**
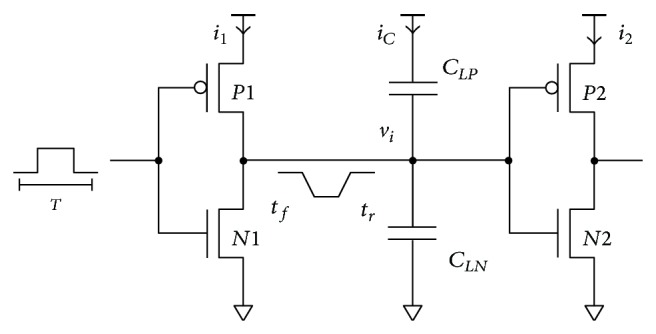
Two-stage CMOS buffer.

A standard two-stage CMOS buffer is depicted in Figure 2. A simplified transistor model (3) [10] is assumed. The model merges transistor geometry and technology parameters into factor *β* = (*μɛ*/*t*_*ox*_)(*w*/*l*), where *μ* is surface mobility of the carriers, *ɛ* and *t*_*ox*_ are permittivity and thickness of the gate insulator, and *w* and *l* are transistor channel's width and length, respectively. Transistor capacitances are represented by *C*_*LP*_ and *C*_*LN*_. Consider

(3) $$\begin{array}{c} i_{d}=\left\{\begin{array}{cc} 0 & v_{gs}\leq V_{T}\\ \beta \left(\left(v_{gs}-V_{T}\right)v_{ds}-\frac{v_{ds}^{2}}{2}\right) & v_{ds}\leq v_{gs}-V_{T}\\ \frac{\beta }{2}{\left(v_{gs}-V_{T}\right)}^{2} & v_{ds}>v_{gs}-V_{T}. \end{array}\right. \end{array}$$

If input signal rise and fall times are zero, then no direct-path current is present in the first stage. Dynamic charge (4) charging and discharging capacitive loads *C*_*LP*_ and *C*_*LN*_ represents the only consumption in the first stage. Consider

(4) $$\begin{array}{c} Q_{d}=\overset{t_{0}+T}{\underset{t_{0}}{\int }}\left(i_{1}+i_{C}\right)dt=\left(C_{LP}+C_{LN}\right)V_{DD}. \end{array}$$

By solving the Kirchhoff's current law for the internal node, input rise and fall times for the second stage can be obtained. They can be approximated by (5) [11]. Consider

(5) $$\begin{array}{c} i_{dN1}+C_{LP}{\left(v_{i}-V_{DD}\right)}^{\prime }+C_{LN}v_{i}^{\prime }=0,\\ i_{dP1}+C_{LP}{\left(v_{i}-V_{DD}\right)}^{\prime }+C_{LN}v_{i}^{\prime }=0,\\ t_{f}=k_{2}\frac{C_{LP}+C_{LN}}{\beta_{N1}V_{DD}},\;\;t_{r}=k_{3}\frac{C_{LP}+C_{LN}}{\beta_{P1}V_{DD}}. \end{array}$$

Constants *k*_2_ and *k*_3_ are equal for *V*_*TN*_ = −*V*_*TP*_.

The second stage has no load. Internal voltage is directly transferred to the output. Therefore, no dynamic consumption is present in the second stage. Since rise and fall times (5) of the second stage input signal are not zero, direct-path current flows during the transition resulting in charge:

(6) $$\begin{array}{c} Q_{s}=\overset{t_{0}+T}{\underset{t_{0}}{\int }}i_{2}\;dt. \end{array}$$

With a linear approximation of the internal voltage transitions during rise and fall times (5), the static charge can be calculated as

(7) $$\begin{array}{c} Q_{s}=\frac{\beta_{N2}}{6V_{DD}}\frac{{\left(V_{DD}+V_{TP}-V_{TN}\right)}^{3}}{{\left(1+\sqrt{\beta_{N2}/\beta_{P2}}\right)}^{2}}\left(t_{r}+t_{f}\right). \end{array}$$

Because there is no capacitive load, no delay is added in the second stage. The low-to-high and the high-to-low output delays are proportional to *t*_*f*_ and *t*_*r*_, respectively. Consider

(8) $$\begin{array}{c} t_{d\mathrm{LH},\text{CMOS}}=k_{4}t_{f},\;\;t_{d\text{HL},\text{CMOS}}=k_{5}t_{r}. \end{array}$$

If the second stage is symmetric, *k*_4_ = *k*_5_ = 1/2.

Assuming *V*_*TN*_ = −*V*_*TP*_ = *V*_*T*_ and symmetric first stage *β*_*N*1_ = *β*_*P*1_ = *β*_1_, the rise and the fall times *t*_*r*_, *t*_*f*_  (5) are equal. Additional symmetry in the second stage *β*_*N*2_ = *β*_*P*2_ = *β*_2_ simplifies the *Q*(*T*_*p*,CMOS_) relation into

(9) $$\begin{array}{c} Q=Q_{d}+Q_{s}=\left(\frac{2\beta_{1}V_{DD}^{2}}{k_{2}}+\frac{\beta_{2}}{6V_{DD}}{\left(V_{DD}-2V_{T}\right)}^{3}\right)T_{p,\text{CMOS}}. \end{array}$$

The propagation delay can be expressed as

(10) $$\begin{array}{c} T_{p,\text{CMOS}}=t_{d\mathrm{LH},\text{CMOS}}=t_{d\text{HL},\text{CMOS}}=k_{2}\frac{C_{LP}+C_{LN}}{2\beta_{1}V_{DD}}. \end{array}$$

The propagation delay is generated by the first stage (*β*_1_) and the second stage gate capacitances. The static and the dynamic consumption increases linearly with *T*_*p*,CMOS_.

## 4. Bidirectional Delay Implementation

**Figure 3:**
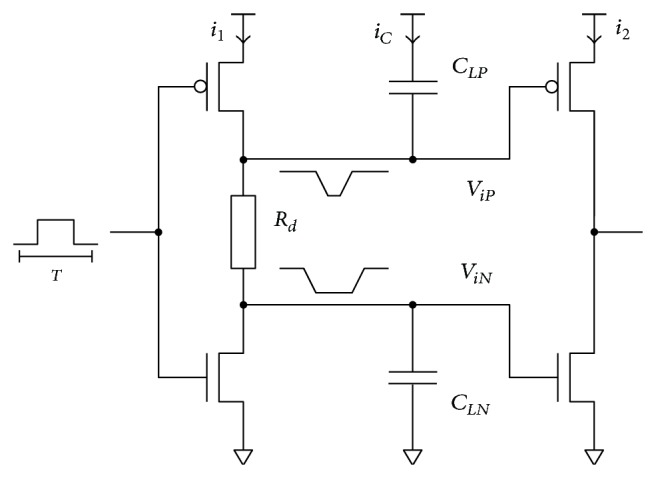
Two-stage BBM buffer with RC delay implementation.

In the simplest case, the bidirectional delay can be implemented with a single resistor *R*_*d*_ (Figure 3), which in combination with the capacitances of the next stage provides the isolation time.

The dynamic charge (4) required for charging and discharging capacitive loads *C*_*LP*_ and *C*_*LN*_ remains the only cause for the power consumption in the first stage.

Internal node voltage *v*_*iP*_, *v*_*iN*_ transients are required for computing the isolation times. Equations (11) and (12) must be solved to obtain *t*_*df*_, *t*_*if*_ and *t*_*dr*_, *t*_*ir*_, respectively. Consider

(11) $$\begin{array}{c} i_{dN1}+C_{LP}{\left(v_{iP}-V_{DD}\right)}^{\prime }+C_{LN}v_{iN}^{\prime }=0,\\ v_{iN}-v_{iP}=R_{d}C_{LP}{\left(v_{iP}-V_{DD}\right)}^{\prime }, \end{array}$$

(12) $$\begin{array}{c} i_{dP1}+C_{LP}{\left(v_{iP}-V_{DD}\right)}^{\prime }+C_{LN}v_{iN}^{\prime }=0,\\ v_{iP}-v_{iN}=R_{d}C_{LN}v_{iN}^{\prime }. \end{array}$$

The solution of the equations is complicated and is not appropriate for manual calculation.

Discharging load capacitances *C*_*LP*_ and *C*_*LN*_ can be dealt with separately in (11) assuming high *R*_*d*_. *R*_*d*_ → *∞* causes *v*_*iP*_′ → 0 and *C*_*LN*_ is discharged first. Influence of *C*_*LP*_ current is negligible. Equation (11) simplifies into *i*_*dN*1_ + *C*_*LN*_ *v*_*iN*_′ = 0. The same deduction holds for charging load capacitances *C*_*LP*_ and *C*_*LN*_ in (12). *R*_*d*_ → *∞* causes *v*_*iN*_′ → 0 and (12) simplifies into *i*_*dP*1_ + *C*_*LP*_(*v*_*iP*_ − *V*_*DD*_)′ = 0. Delay times *t*_*df*_ and *t*_*dr*_ can be obtained by solving the simplified versions of (11) and (12) as in (5)

(13) $$\begin{array}{c} t_{df}=k_{6}\frac{C_{LN}}{\beta_{N1}V_{DD}},\;\;t_{dr}=k_{7}\frac{C_{LP}}{\beta_{P1}V_{DD}}. \end{array}$$

The isolation time depends on the RC constant. Consider

(14) $$\begin{array}{c} t_{if}=k_{8}R_{d}{\text{C}}_{LP},\;\;t_{ir}=k_{8}R_{d}C_{LN}. \end{array}$$

Constants *k*_6_ and *k*_7_ are equal for *V*_*TN*_ = −*V*_*TP*_.

Since node voltages *v*_*iP*_ and *v*_*iN*_ are time skewed, static consumption *Q*_*s*_ in the second stage is zero. The total consumption is therefore equal to the dynamic consumption in the first stage (4). Assuming *V*_*TN*_ = −*V*_*TP*_ = *V*_*T*_ and symmetric first stage *β*_*N*1_ = *β*_*P*1_ = *β*_1_, the propagation delay (1) can be expressed as

(15) $$\begin{array}{c} T_{p}=\left(\frac{k_{6}}{\beta_{1}V_{DD}}+k_{8}R_{d}\right)\frac{C_{LP}+C_{LN}}{2}, \end{array}$$

which can be used to obtain

(16) $$\begin{array}{c} Q=\frac{2\beta_{1}V_{DD}^{2}}{k_{6}+k_{8}R_{d}\beta_{1}V_{DD}}T_{p}. \end{array}$$

Charge consumption again linearly increases with propagation delay.

To ensure rise and fall delay symmetry *t*_*dLH*⁡_ = *t*_*df*_ + *k*_9_ *t*_*if*_ = *t*_*d*HL_ = *t*_*dr*_ + *k*_10_ *t*_*ir*_, a balance among variables in (13) and (14) is required. If both stages are symmetric and *V*_*TN*_ = −*V*_*TP*_, then the second stage gate capacitances must also be equal (*C*_*LP*_ = *C*_*LN*_). Capacitances *C*_*LP*_ and *C*_*LN*_ depend only on the gate capacitance in the first approximation. The condition *C*_*LP*_ = *C*_*LN*_ can be met by increasing channel length of the second stage NMOS transistor, which degrades its driving performance. Yet another way is to assume that capacitive load is composed of gate capacitance and various stray capacitances (*C*_*L*_ = *C*_gate_ + *C*_stray_). Smaller NMOS gate can be partly compensated by adding more parasitic capacitance to the NMOS gate. Larger parasitic NMOS stray capacitance can be introduced by different gate connections in case the layout allows such modifications.

The analysis above holds if *R*_*d*_ is high enough and static consumption *Q*_*s*_ is consequently negligible. The isolation slope must end before the information slope begins. In the first approximation conditions,

(17) $$\begin{array}{c} k_{2}\frac{C_{LN}}{\beta_{N1}V_{DD}}<k_{11}R_{d}C_{LP},\;\;k_{3}\frac{C_{LP}}{\beta_{P1}V_{DD}}<k_{11}R_{d}C_{LN} \end{array}$$

must be fulfilled, leading to

(18) $$\begin{array}{c} R_{d}>\max\left(\frac{k_{2}C_{LN}}{k_{11}C_{LP}\beta_{N1}V_{DD}},\;\;\frac{k_{3}C_{LP}}{k_{11}C_{LN}\beta_{P1}V_{DD}}\right). \end{array}$$

**Figure 4:**
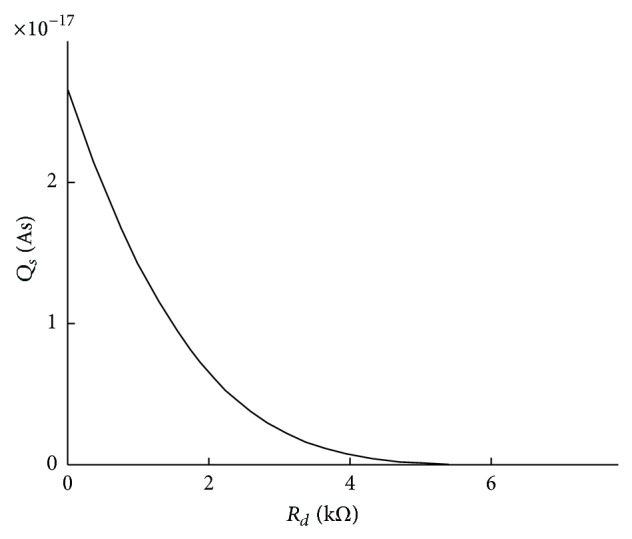
*Q*_*s*_(*R*_*d*_) dependence obtained with SPICE simulation for *V*_*TN*_ = 0.5 V, *V*_*TP*_ = −0.5 V, *KP*_*N*_ = 350 *μ*A/V^2^, *KP*_*P*_ = 90 *μ*A/V^2^, *C*_*ox*_ = 8.6 mAs/Vm^2^, *V*_*DD*_ = 1.8 V, *w*_*P*1_ = 860 nm, *l*_*P*1_ = 180 nm, *w*_*N*1_ = 220 nm, *l*_*N*1_ = 180 nm, *w*_*P*2_ = 310 nm, *l*_*P*2_ = 180 nm, *w*_*N*2_ = 220 nm, and *l*_*N*2_ = 250 nm.

The dependency of *Q*_*s*_ on *R*_*d*_ is depicted in Figure 4.

Although the concept of introducing a bidirectional delay using *R*_*d*_ can be expanded to logic gates, the overhead of increased delay combined with double wiring hardly justifies the energy savings. This approach shows its advantage in circuits with productive use of delay, such as edge-triggered storage elements and clock distribution networks.

## 5. Optimization Problem

Analysis of the circuit in Figure 3 shows that obtaining a specific delay *t*_requested_ with minimum charge consumption is an optimization problem. Minimum of the function *Q*(*R*_*d*_, **w**, **l**) represent the optimal solution. Vectors **w** and **l** represent transistor channel widths and lengths, which define gain factors (*β*) and capacitances. The implicit constraint *t*_*dLH*⁡_ = *t*_*d*HL_ = *t*_requested_ is imposed on the solution.

**Figure 5:**
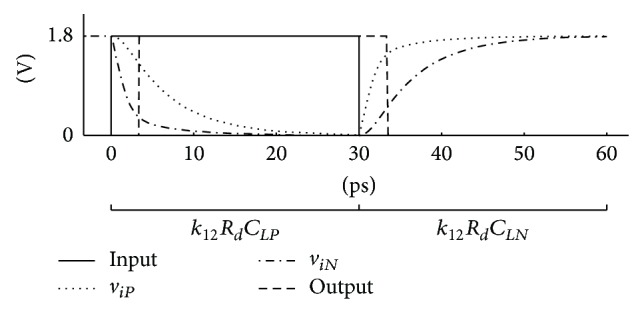
Transient phenomena obtained with SPICE simulation for *R*_*d*_ = 10 k*Ω*.

Delay implementation with *R*_*d*_ causes long charging phases of *C*_*LP*_ and *C*_*LN*_. Transient phenomena of the information slope may not be concluded before the transistors in the next stage switch state (Figure 5). The input signal must remain constant during the transient. Otherwise the next delay is shortened. For this reason another implicit constraint defining the maximum length of the transient is introduced into the optimization problem *k*_12_ *R*_*d*_ *C*_*LP*_ < *t*_max⁡_ and *k*_12_ *R*_*d*_ *C*_*LN*_ < *t*_max⁡_. The input signal must stay constant for at least *t*_max⁡_ after every transition.

The manual calculation is derived from a simplified static transistor model (3). Dynamic behavior is modeled with constant gate capacitances *C*_*LP*_ and *C*_*LN*_. These capacitances are voltage dependent. The optimization procedure of a real world BBM buffer must consider numerous higher order effects that were neglected in the first approximation, such as:

- input signal is not an ideal rectangular shape voltage generator,
- output load is not zero,
- the MOSFET should include model with higher order static (channel-length modulation, short-channel effect, sub-threshold conductivity, etc.) and dynamic (nonlinear capacitances, etc.) effects,
- parasites (layout, wiring, etc.) have to be taken into account.

## 6. Transistor-Based Delay Implementation

**Figure 6:**
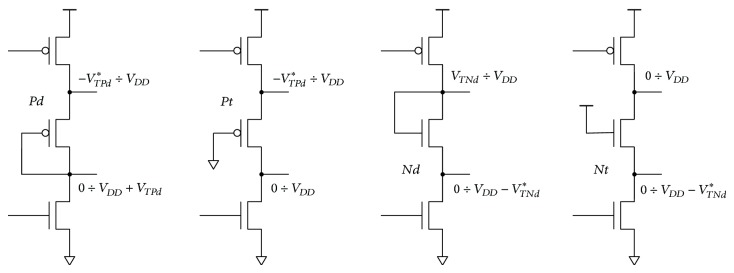
Single transistor delay implementations (^*^the threshold voltage modified due to the body effect). Transistors Pd and Nd are connected as diodes while transistors Pt and Nt are connected as triodes.

*R*_*d*_ implementation with high-resistance polysilicon is area consuming and poorly controlled. One or more MOS transistors can be used instead. If a single delay transistor is connected as a diode or a triode (Figure 6), then the output voltage swing is reduced. The voltage drop is defined by the threshold voltage of the delay transistor *V*_*Td*_. Voltage swing reduction applies to one or both outputs depending on the configuration. This has several implications. The dynamic charge *Q*_*d*_  (4) is reduced from *C*_*L*_ *V*_*DD*_ to *C*_*L*_ *V*_swing_ consequently reducing the power consumption. This causes the delay to increase due to transistor's high resistance in the saturation region and results in long transient phenomena in the information slope. On the other hand, the lower voltage swing is required for reaching the threshold voltage of the next stage, which in turn decreases the delay. A fairly high supply voltage *V*_*DD*_ > 5*V*_*Td*_ is required. Long information transients and high supply voltage make the reduced swing topologies inappropriate.

**Figure 7:**
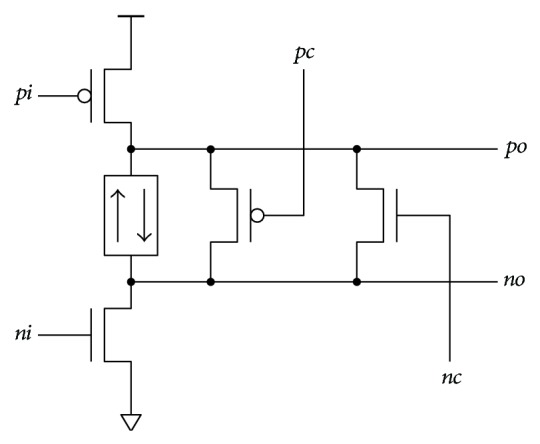
Level restoration.

Full-swing can be achieved with additional level restoration transistors (Figure 7). The PMOS (NMOS) level restoration transistor restores the high (low) level. Level restoration transistor(s) can be combined in parallel with any delay element from Figure 6. Controlling signals *pc*, *nc* are delayed input signals *pi*, *ni*, which can for instance be obtained at the next stage output.

**Figure 8:**
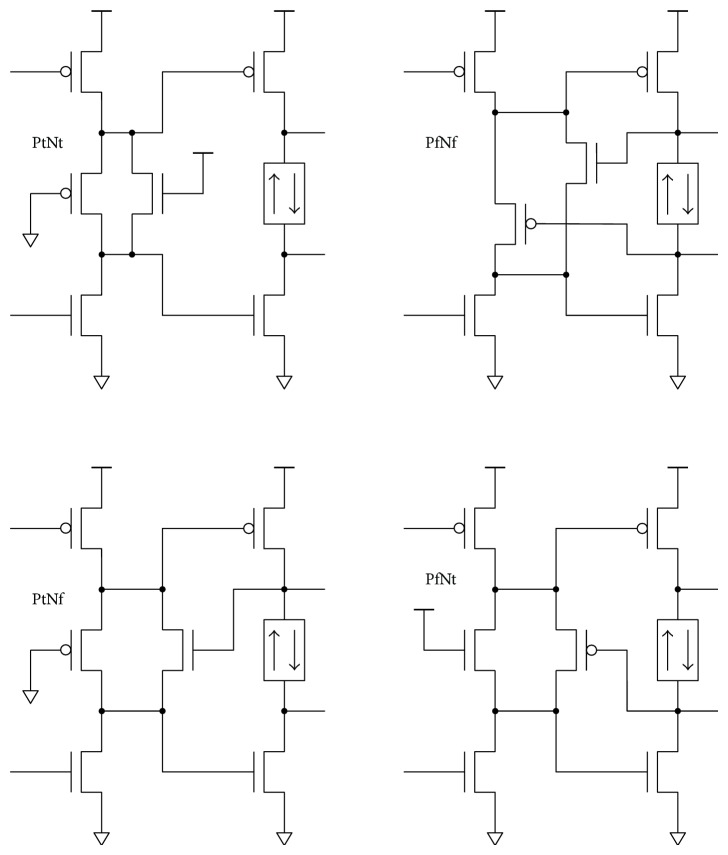
Two transistor full-swing delay implementations.

On gate level, every additional component introduces its own parasitic capacitances causing additional power overhead that must be justified. Therefore, the number of transistors must be kept as low as possible. At least two transistors are needed for full-swing delay implementation. Possible topologies are shown in Figure 8. There are four combinations of PMOS and NMOS delay transistors in triode mode (PtNt), level restoration transistors without delay transistors (PfNf), and PMOS or NMOS delay transistor with appropriate level restoration transistor (PtNf and PfNt). Controlling level restoration signals (*pc*, *nc*) is taken from the next stage output. Using feedback for level restoration is a logical choice, since level restoration is required immediately after the next stage switches state.

## 7. Noninverting Delay Cell

The dual-ramp (i.e., BBM) CMOS inverter is well suited for building low-power low-precision delay elements. It conveniently combines the delay with short-circuit current elimination. Generally, the delay circuit can be constructed as cascade of several BBM stages comprising elements depicted in Figure 1. In the simplest case the circuit can be reduced to the interfacing elements depicted in Figures 1(a) and 1(c). This results in a 6-transistor noninverting delay cell when full-swing delay topologies from Figure 8 are used.

**Figure 9:**
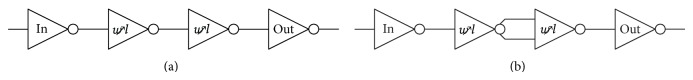
Standard (a) and proposed (b) simple dual-ramp (i.e., BBM) delay cell.

To verify the proposed principle, all four variations of the simple dual-ramp delay cell were compared to a standard serial connection of two CMOS inverters. All five delay circuits were sized for smallest possible charge consumption at a required delay. Digital cell sizing, including delay, is highly dependent on a required fan-in and fan-out properties. To eliminate this dependence, standard input and output unit inverters were added, defining equal fan-in and fan-out properties (Figure 9) for delay circuits. Both inverters contribute to the delay and are considered as part of the cell. The final sizing (i.e., optimization result) of a delay circuit is of course tailored to the selected pair of input and output standard unit inverters. Standard CMOS and dual-ramp (BBM) delay cells with input and output buffers are shown in Figure 9.

## 8. Results

Sizing cells from Figure 9 to a required delay is an eight- or a twelve-dimensional optimization problem. Finding the global minimum is not a trivial task, especially if there are plenty of local minima. Therefore, every optimization run was repeated several times in various parts of the parameter space until the global minimum was confirmed.

A parallel version of SADE [12] global optimization method was used. The optimization procedure ran in parallel on a cluster of 100 computing nodes driven by the PyOPUS [13] library. 25 Intel Core i5 2.66 GHz processors (4 nodes per processor) were used. Circuit simulations were performed by the Synopsys HSPICE circuit simulator with the TSMC 0.18 *μ*m/1.8 V process parameters.

Beside transistor sizing, the delay and power dissipation also depend on the circuit layout. Automatic layout procedure and extracting parasitic node capacitances should be done in every iteration before the simulation. The authors could not include the layout and extraction steps into the optimization loop due to not small, but, nevertheless, limited computer power. Therefore, the node capacitances due to layout were not taken into account. To approximate the real conditions, transistor parasitics due to the connection geometry were included. Layout rules (*A*_*d*_ = *A*_*s*_ = 0.8 *μ*m × *w*, *P*_*d*_ = *P*_*s*_ = 1.5 *μ*m + *w*) were applied. But, in spite of described imperfection, the obtained results for standard and proposed delay cell still indicate the capabilities of the two topologies.

Straightforward sizing of the cells produces inappropriate results. Optimizer finds a sizing with small consumption and a perfect delay match. These are in fact degenerated circuits whose operation depends on poorly defined parasitics causing very long internal transients (Figure 5). To obtain usable solutions based on well-defined manufacturing process parameters, such as gate capacitance and intrinsic transconductance, additional implicit constraints were required.

The first set of safeguarding constraints avoids extreme over- and undershoots thus preventing circuit operation based on parasitic capacitances (e.g., Miller capacitance). Miller capacitances of large transistors are the main source of the delay time. In that unwanted case, the delay results from charging and discharging the parasitic capacitances.

The second set of constraints ensures that the steady state is reached after *t*_max⁡_ (Figure 5). Very long internal transients with smaller charge consumption are otherwise superior from the optimization point of view.

The third set represents additional requirements needed to obtain noise resistant circuits. Noise margins are obtained by requiring stable steady state node voltages during *p*- and *n*-substrate potential disturbances.

**Figure 10:**
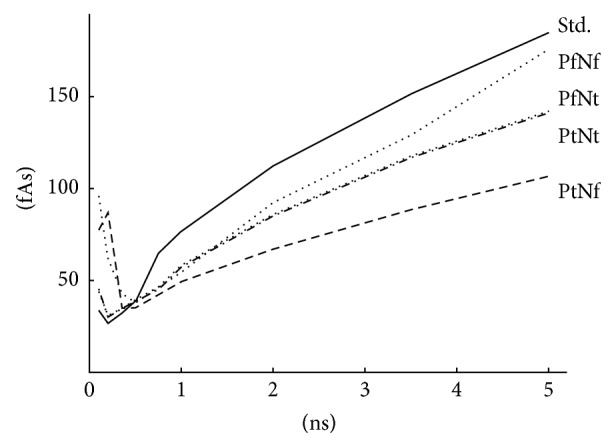
Dependence of charge consumption on the delay.

Figure 10 illustrates the results summarized in Table 1. Each topology was sized targeting delays from 100 ps to 5 ns. Charge consumption growth with delay becomes approximately linear for delays above 1 ns as (9) and (16) predict. The topology with PMOS transistor in triode mode and NMOS level restoration transistor (PtNf) turns out as the most efficient. In comparison with the standard topology, charge savings are slightly higher than 40%.

**Figure 11:**
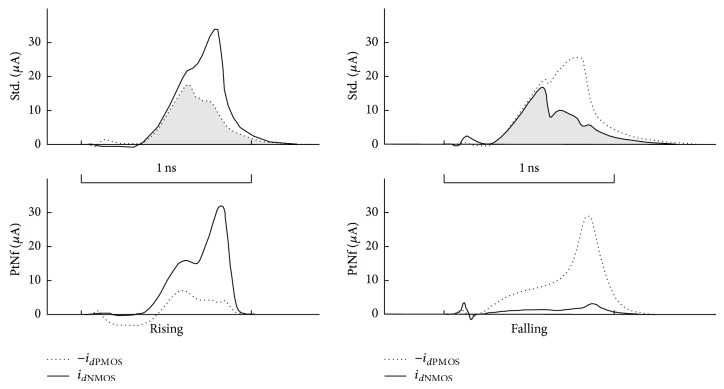
Third stage drain currents obtained with SPICE simulation for *T*_*p*_ = 750 ps (sizing: std. *w*_*P*1_ = 220 nm, *l*_*P*1_ = 370 nm, *w*_*N*1_ = 220 nm, *l*_*N*1_ = 880 nm, *w*_*P*2_ = 880 nm, *l*_*P*2_ = 730 nm, *w*_*N*2_ = 1.05 *μ*m, *l*_*N*2_ = 1.32 *μ*m; PtNf *w*_*P*1_ = 220 nm, *l*_*P*1_ = 740 nm, *w*_*N*1_ = 390 nm, *l*_*N*1_ = 180 nm, *w*_*Pt*_ = 220 nm, *l*_*Pt*_ = 350 nm, *w*_*Nf*_ = 220 nm, *l*_*Nf*_ = 950 nm, *w*_*P*2_ = 220 nm, *l*_*P*2_ = 180 nm, *w*_*N*2_ = 220 nm, and *l*_*N*2_ = 220 nm).

Elimination of static consumption can be observed in the third stage. It is the only stage actually driven by time-skewed signals. Drain currents for *T*_*p*_ = 750 ps sizing are depicted in Figure 11. Similar current transients can be observed with other BBM topologies and delays. The large direct-path current in the standard topology (shadowed) is almost completely eliminated in the PtNf topology. The optimization procedure obtained the required delay with large channel lengths in the second stage. This means that the bulk of the delay is caused by the third stage gate capacitances.

## 9. Applications

**Figure 12:**
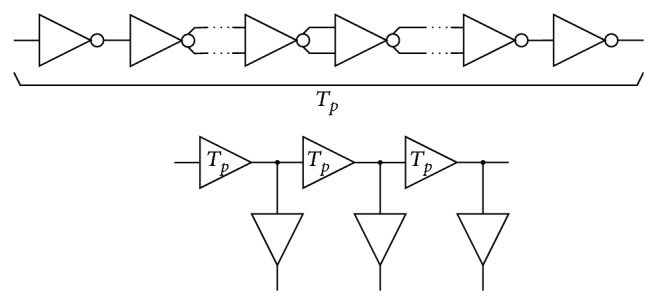
Delay line implemented with BBM delay dells.

Dual-ramp BBM delay cells can be used for constructing low-power delay lines. All that needs to be done is to replace standard delay elements with proposed ones, as shown in Figure 12. The number of stages in one delay element *T*_*p*_ can vary depending on the required delay.

**Figure 13:**
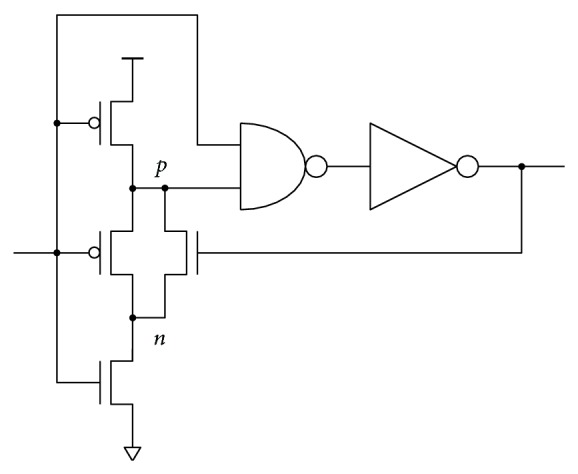
BBM based leading-edge pulse generator.

The BBM inverter with level restoration can be used as a key element in a leading-edge pulse generator (Figure 13). Pulse generators are frequently used for generating precharge or data-strobe pulses in dynamic logic and flip-flops. Since, in this case, the delay is needed only for the low-to-high transition, the circuit can be simplified. A single BBM stage provides enough delay for the AND-type edge detection. The width of the generated pulse is defined by the AND gate delay and the parasitic capacitance of node *p* combined with the resistance of the discharging NMOS feedback transistor. The inherent delay of the output pulse dictated by the AND gate provides enough time to reset node *p* through the NMOS feedback transistor. The voltage level of node *n* is restored through the precharging PMOS transistor. In this context, the BBM inverter acts as a feedback switch with limited impact on the delay.

The BBM topology in Figure 13 was compared to standard leading-edge pulse generator with various delay line lengths. Low-power cells (inverter and NAND gate) from industry standard library were used. Input and output buffers were added to equalize fan-in and fan-out properties. Manufacturing process layout rules defining transistor geometry were applied during the sizing procedure (i.e., optimization). And of course some previously described constraints were essential to obtain sensible results.

The results are summarized in Table 2. The pulse width, the total charge consumption, and the delay line charge consumption of the standard leading-edge pulse generator with 3, 5, 7, 9, and 11 cascaded inverters in the delay were measured first. Then, the BBM topology was sized to the individual pulse widths. The charge consumptions of the equivalent BBM based leading-edge pulse generators were obtained. The simplicity of the delay implementation saves up to 50% of the total switching energy compared to the standard realization. The advantage of the BBM based circuit is clearly presented when the charge consumption of the delay line is measured separately. For standard implementation, the consumption linearly increases with the number of inverters in the delay. On the other hand, the consumption of the BBM inverter is almost constant. This is due to the constant number of transistors. A slight increase can be observed for longer pulse widths, which is caused by higher parasitic capacitances of larger transistors required in that case. Note that the consumption of the inverter supplying the feedback is included only in the total charge consumption measurement.

**Figure 14:**
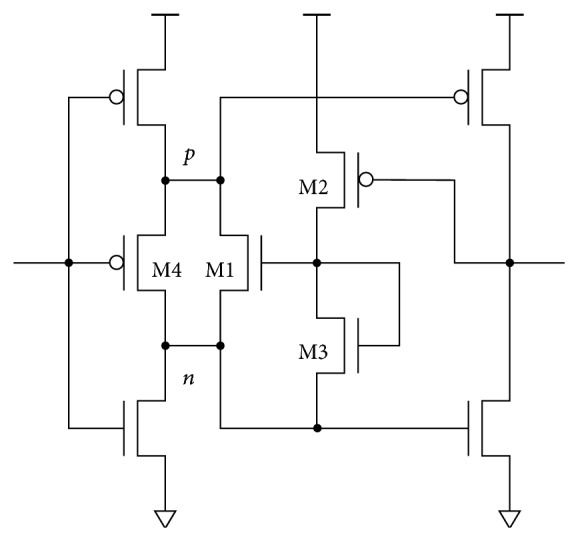
BBM delay cell with biased NMOS current discharge.

**Figure 15:**
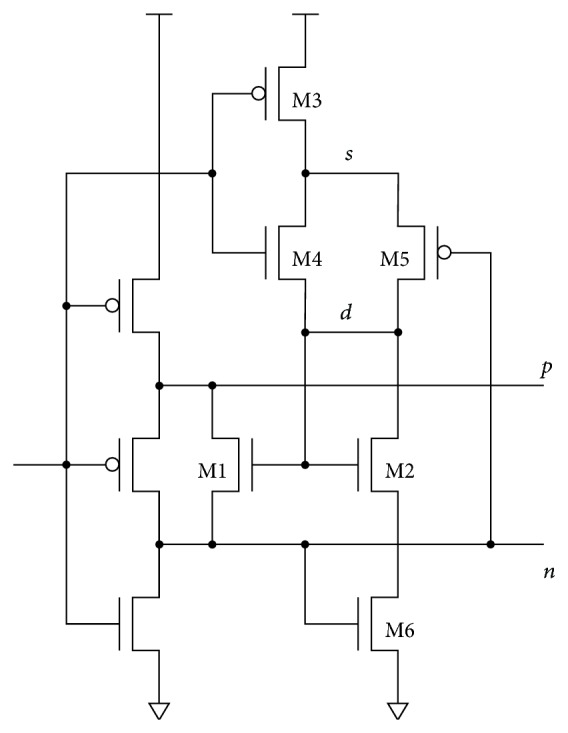
Dynamically biased delay cell.

Two potential topology modifications not requiring the feedback are given in Figures 14 and 15. The AND gate delay therefore does not affect the width of the generated pulse. The price for removing the feedback is an increased number of transistors, which causes higher charge consumption compared to the feedback implementation from Figure 13.

The delay is defined more precisely if the discharge current is controlled by a biased MOS transistor [14] (Figure 14). The leading-edge delay of the input signal is defined by the time needed for discharging the parasitic capacitance of node *p* through the NMOS feedback transistor M1. Transistor M4 presets node *n* before the delay transient, providing the inverted input signal. The latter is combined with the delayed output signal for switching the current through M2/M3 when the biasing voltage on the M1 gate is needed [15].

The DC power consumption of the biasing circuit can be reduced by dynamic biasing presented in Figure 15. When the input signal is low, the circuit prepares the initial conditions for the delay transient: the voltage of node *d* is clamped by M2 to *V*_*TN*_ and node *s* is precharged through M3 to *V*_*DD*_. In the active phase, when input goes to high, node *d* is charged by the parasitic capacitance of node *s* through the transmission gate M4/M5, thus raising the M1 gate voltage to the desired level for the delay transient. The effective voltage at the M1 gate is given by *V*_*d*_ = (*V*_*DD*_ *C*_*s*_ + *V*_*TN*_ *C*_*d*_)/(*C*_*s*_ + *C*_*d*_), where *C*_*s*_ and *C*_*d*_ are the parasitic capacitances of nodes *s* and *d*, respectively. Parasitic capacitances can be trimmed by adding diffusion areas to the relevant transistors or using gate oxide capacitors. The transistors in the switching network (M2*⋯*M6) are minimum sized.

## 10. Conclusion

A modified static CMOS inverter has been presented which reduces direct-path current in circuits, where the delay is a required part of the circuit's functionality. The proposed BBM inverter is well-suited for building low-power low-precision delay elements due to its ability to combine delay and direct-path current elimination in one single stage. The suppression is based on the serially connected delay element in the inverter output thus providing time-skewed output signals. Two output signals provide additional capabilities for compact functional solutions. The principle of operation has been verified by performing delay cell optimizations for various delay element implementations. With the exception of very short delays, the proposed BBM inverter structure improves the power budget compared to the standard cascaded inverter transport delay implementation. Besides the delay lines, variations of the proposed topology can be used in other slow transition circuits. Edge-detector circuit featuring BBM topology has been presented.

## Figures and Tables

**Table 1:** Charge consumption results for the standard (std.) and the BBM delay cells depicted in Figure 9 measured for one rising and one falling slope. The columns denoted by the percent sign represent the percentage of the standard delay cell's consumption.

*T*_*p*_ [ns]	std. [fAs]	PtNt [fAs]	[%]	PtNf [fAs]	[%]	PfNt [fAs]	[%]	PfNf [fAs]	[%]
0.1	34^*^	45^*^	—	78^*^	—	44^*^	—	97^*^	—
0.2	26	30	115	87^*^	—	30	115	62^*^	—
0.35	32	34	106	34	106	35	109	42	131
0.5	39	38	97	35	90	39	100	38	97
0.75	65	45	69	42	65	46	71	46	71
1.0	77	58	75	49	64	58	75	55	71
2.0	113	86	76	67	59	86	76	93	82
3.5	153	118	77	89	58	118	77	130	85
5.0	186	142	76	108	58	143	77	177	95

^*^Delay target was not achieved.

**Table 2:** Charge consumption results for the standard and the BBM based leading-edge pulse generator depicted in Figure 13 measured for one input impulse. The columns denoted by the percent sign represent the percentage of the standard realization's consumption.

Standard				BBM based			
Number of inv.	Width [ps]	Total [fAs]	Delay [fAs]	Total [fAs]	[%]	Delay [fAs]	[%]
3	200	171	56	143^*^	—	17^*^	—
5	330	210	93	142	68	16	17
7	450	247	130	147	60	16	12
9	570	284	167	153	54	16	10
11	690	321	204	161	50	17	8

^*^Pulse width target was not achieved.
